# Thickness of orthodontic clear aligners after thermoforming and after 10 days of intraoral exposure: a prospective clinical study

**DOI:** 10.1186/s40510-019-0289-6

**Published:** 2019-09-09

**Authors:** Rosaria Bucci, Roberto Rongo, Carmine Levatè, Ambrosina Michelotti, Sandro Barone, Armando Viviano Razionale, Vincenzo D’Antò

**Affiliations:** 1 Department of Neurosciences, Reproductive Sciences and Oral Sciences, University of Naples Federico II, via Pansini 5, 80131 Naples, Italy; 2 Department of Civil and Industrial Engineering, University of Pisa, Largo Lucio Lazzarino 1, 56122 Pisa, Italy

**Keywords:** Orthodontic appliance, Clear aligners, Thermoplastic, Mechanical proprieties, Dimensional stability, Intraoral ageing, Thermoforming, Aesthetic, Adult

## Abstract

**Background:**

Clear aligners (CA) are among the most chosen orthodontic therapies for patients who require an invisible treatment. Previous studies showed that the thermoforming process and the complexity of the intraoral environment might alter the properties of these devices. The aim of the current prospective clinical study was to assess the thickness changes of the CA after 10 days of intraoral use. The secondary aim was to assess the reproducibility of the thermoforming process, in terms of aligner thickness.

**Materials and methods:**

CA from 18 consecutive patients (13 women, 5 men, mean age 28.8 ± 9.6 years) were investigated. Before intraoral exposure (T0), the thickness of the unused CA was measured at different occlusal points on a 3D model with a dedicated software (Geomagic Qualify 2013; 3D Systems, Rock Hill, SC, USA). Two CA configurations were studied: passive maxillary aligner (P—no tooth movement; no shape for attachments) and active maxillary aligner (A—tooth movement; shape for attachments and divot). The used aligners were returned after 10 days (T1) and the thickness measurements were repeated. A Student’s *t* test for paired data (T1 vs. T0) was applied to compare the thicknesses of used and unused devices (significance level after Bonferroni correction for multiple comparison was set at *p* < 0.0014). Furthermore, to study the reproducibility of the thermoforming process, P and A aligners were thermoformed twice, and the thicknesses of the two unused thermoformed devices were compared by means of Student’s *t* test for paired data (significance level after Bonferroni correction for multiple comparison was set at *p* < 0.0014) and Dahlberg’s error.

**Results:**

The thermoforming process showed good reproducibility for both aligner configurations, with a maximum Dahlberg’s error of 0.13 mm. After intraoral use, the thickness of P showed some statistically significant, but not clinically relevant, thickness changes as compared to the unused aligners, while A did not show any significant changes.

**Conclusion:**

Considering the thickness changes, the thermoforming process is reliable both with active and passive aligner configurations. Also, the CA examined show good thickness stability after physiological intraoral ageing in a population of healthy adults.

## Background

In the last decades, the role of aesthetics in patients’ decisions to receive orthodontic treatment has crucially increased [1]. A growing number of adolescent and adult patients require aesthetic treatments that are compatible with their daily lives and do not compromise their quality of life [2–4]. While orthopaedic and functional appliances still represent the standard of care for the management of skeletal discrepancies in paediatric patients [5–7], clear aligners (CA) have been introduced as valid aesthetic alternative to fixed brackets for the correction of mild to moderate malocclusions, due to their satisfactory aligning and levelling results [8, 9]. Besides, the use of CA resulted in shorter treatment duration and chair-side time when compared to conventional fixed therapy with braces and archwires [10].

The valuable progress in the aligners-attachments interaction [11] and the possibility to combine CA with auxiliaries, such as skeletal anchorage or orthognathic surgery, allowed for the resolution of more complex cases thus extending the spectrum of cases treatable with orthodontic aligner therapy [12]. Also, the implementation of the biomaterials used for the fabrication of the CA has led to a constant improvement in the performances of these appliances [13].

Numerous previous researches studied the mechanical and aesthetic properties of biomaterials for their application in the orthodontic field [14–16]. Nowadays, thermoplastic materials are widely used for the fabrication of CA due to their excellent characteristics [15, 17, 18]. In particular, polyester, copolyester, polycarbonate, thermoplastic polyurethanes and polypropylene are the dominant thermoplastic material mixtures used for the manufacture of CA [19]. These materials allow the fabrication of highly precise devices via a thermoforming process on accurate models of patients’ malocclusions. However, studies in a simulated intraoral environment and on specimens of retrieved aligners after intraoral exposure pointed out that these devices do not maintain their original shape or composition in the mouth. In fact, temperature, humidity, salivary enzymes and elastic deformation might influence the properties of CA, suggesting that the mechanical behaviour of dental thermoplastic materials varies due to environmental factors [13, 20–22]. Therefore, since CA are generally recommended to be used for 7–14 days in each stage, the progressive change of the mechanical properties in the intraoral environment might influence the treatment efficacy. In particular, increased stiffness has been found after intraoral use, due to an alteration of the polymer crystallinity [23].

Besides aligner material, some geometric parameters of the aligner, such as the thickness, may influence the magnitude of the forces delivered to the tooth [24]. In fact, the clinical behaviour of the thermoplastic materials is highly influenced by the thickness of the device [25, 26]. Furthermore, changes in the mechanical properties might be related to a variation of aligner shape due to the intraoral ageing. For instance, the wear of the occlusal surface of the aligners has been claimed to influence the duration of the load and of the forces delivered [25]. In addition, previous studies on retrieved aligners reported substantial morphological variations after clinical or in vitro ageing, including adsorption of integuments, microcrack, abrasion at the cusp tips and localised calcification [22, 27]. Therefore, a critical understanding of the geometrical variations of the different thermoplastic materials and of the effects of the intraoral ageing is essential to plan the correct sequencing of tooth movement and to achieve the desired treatment outcome.

Systematic multiscale analysis has been used to measure the effect of the thermoforming process on physical and mechanical proprieties of various thermoplastic materials with different thicknesses [28]. Transparency, water absorption, surface hardness, flexure and elastic moduli, and tensile and flexural forces resulted significantly changed after thermoforming, thus suggesting that mechanical properties of thermoplastic materials used for the manufacturing of CA should take into account the thermoforming process in order to study their clinical application [28]. Furthermore, previous studies assessing the effects of the thickness of CA on the clinical performance took into account only the thickness of the original foil used to manufacture the aligner [29–32], neglecting possible changes of thickness related to the thermoforming process.

The primary aim of the present prospective clinical study was to evaluate the thickness changes of the whole occlusal surface of CA after 10 days of full-time intraoral use in adult healthy patients, as compared to unused aligners. Furthermore, the secondary aim was to determine the reproducibility of the thermoforming of CA, in terms of aligner thickness.

## Materials and methods

### Sample

The study sample consisted of 18 consecutive patients, 13 women (mean age ± SD 31.6 ± 10.2 years) and 5 men (mean age ± SD 26.0 ± 9.0 years), referred to the School of Orthodontics of the Department of Neurosciences, Reproductive Sciences and Oral Sciences at the University of Naples Federico II (Italy) between September 2017 and November 2018. The first consultation was performed by one expert operator (VD), to evaluate whether they were suitable for CA orthodontic treatment.

Inclusion criteria for enrolment in the study were the following:

- Subjects > 18 years old, with a complete natural dentition except for the third molars, adequate oral hygiene, no signs and symptoms of periodontal diseases
- Healthy subjects without medical or mental problems
- Subjects who agreed to participate in the study

Subjects were excluded from the study if they presented one of the following exclusion criteria:

- Use of medication for neurological diseases (such as anti-depressants)
- Orofacial pain or temporomandibular pain patients
- Presence of active carious lesions
- Previous orthodontic treatment

### Study materials

The aligners used in this study were made of polyethylene terephthalate glycol copolyester (PET-G) (0.75-mm thick) and were all produced with the same thermoforming machine (Ministar of Scheu Dental, Iserlohn, Germany) using the vacuum thermoforming process with a temperature of 220 °C, by the company AirNivol S.r.l. (Navacchio, Italy). The models used as a mould for the thermoforming of the aligners were obtained by a 3D printing machine (Stratasys—Objet Eden 500). Each aligner was manually cut along gingival margins and the edges were manually refined to ensure the best comfort for the patients and to avoid negative influences that could arise from an automatic cutting process.

Two different aligner configurations were studied: the *passive aligner* (P) and the *active aligner* (A). P was a smooth aligner, which did not deliver any tooth movement and was designed without shape for attachment and/or divot and did not require interproximal reduction; A contained shape for attachments and divots, according to the planned tooth movement.

### Clinical protocol

Firstly, the patients were provided with the P aligner. After 10 days of full-time (22 h/day) intraoral use, they were invited to the clinic to return the used (“retrieved”) P aligner. In the same appointment, attachments were built on the teeth according to the indication of the individual treatment planning, and the patients were provided with the A aligner. After 10 days, they returned the used A aligner. All participants were blinded to the absence of tooth movement in the P aligner.

During the experimental session, the subjects were invited to report the wearing hours of the aligners on a written compliance diary. Individuals who were not satisfactorily compliant with the therapy were excluded from the study of the intraoral ageing.

### Measurement of the thickness of clear aligners

**Fig. 1:**
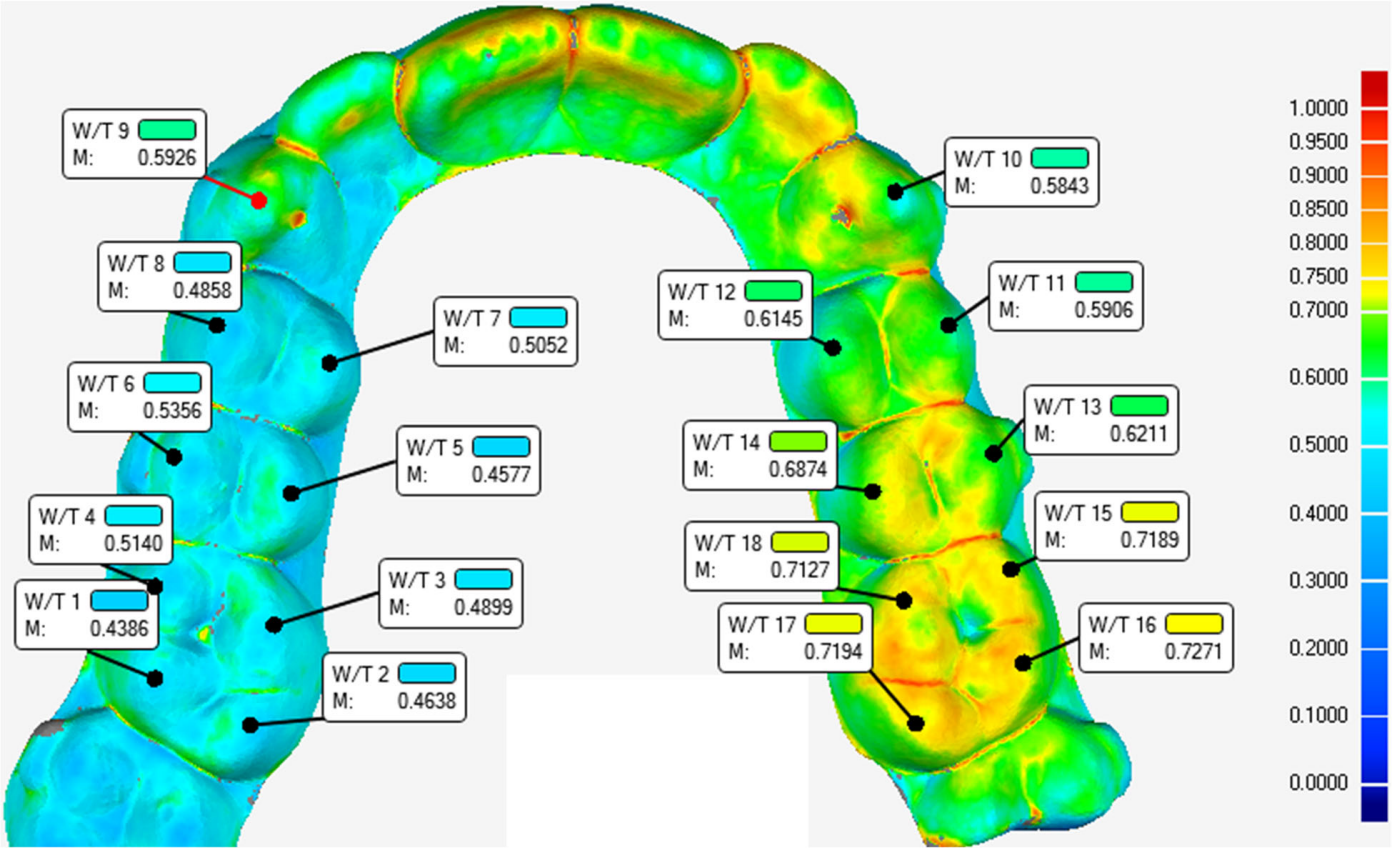
Thickness distribution map with left and right reference points adopted for the measurement of the thickness of the aligners

The thickness of the 18 P and 18 A aligners before intraoral use (“as-received” aligners) (T0) was measured at different reference points using the method described in a paper authored by Barone and co-workers [33]. Briefly, all the aligners were scanned by one operator (AR) with a laboratory optical 3D scanner specifically developed for dental models (Scanner 3Shape E2; Accuracy, 10 μm; Ref. ISO 12836). The scan step is fully automatic and produces as output the whole 3D model of the measured aligner. The obtained digital model was imported in a software aimed at the control quality of optically scanned samples for industrial manufacturing applications (Geomagic Qualify 2013 software; 3D Systems, Rock Hill, SC, USA). The software allows for directly comparing the measured model with its designed CAD counterpart and can extract several measurements from the 3D model. For the purpose of this work, the software was used to measure the whole thickness of the aligners, by one trained operator (CL). The thickness is measured as the normal distance between correspondent points on the internal and on the external surface of the model. Among all the measured points, a limited number of reference points both on the left (L) and right side (R) of the maxillary arch, were selected as follows (Fig. 1):

- U3 (centre of the cuspid of the canine)
- U4B and U5B (centre of the buccal cuspid of the first and second premolars)
- U4P and U5P (centre of the palatal cuspid of the first and second premolars)
- U6MB and U6MP (centre of the mesio-buccal cuspid and mesio-palatal cuspid of the first molars)
- U6DB and U6DP (centre of the cuspid of disto-buccal and disto-palatal cuspid of the first molars)

### Assessment of the dimensional changes after intraoral ageing

After 10 days of full-time intraoral use (T1), the aligners were returned and the thickness measurements were repeated for P and A retrieved aligners.

### Dimensional reproducibility of the thermoforming process

To test the reproducibility of the thermoforming process, P and A aligners (18 P and 18 A) were thermoformed twice (passive2—P2 and active2—A2). The same measurements were performed (72 aligners total) to compare the thickness of the unused thermoformed appliances.

### Method error

The technical errors of measurement were calculated for all the aligners configurations analysed in this study, from 5 randomly selected patients. All measurements were re-assessed by the same operator (CL) after a memory washout period of at least 8 weeks. The method error for all measurements was calculated using Dahlberg formula. Furthermore, systematic differences between duplicated measurements were tested using a paired Student’s *t* test with the type I error set at *p* < 0.05.

### Statistical analysis

A pilot study was performed on three subjects to calculate the sample size of the study. The sample size was computed considering *α* = 0.0014 (Bonferroni correction for multiple comparisons 0.05/36), power = 0.80, an effect size of 1.1 considering an average reduction of the thickness due to intraoral ageing of 0.09 mm and a standard deviation 0.08 for the U6DB. Hence, a sample size of at least 17 patients was determined to be adequate comparing the groups with a paired *t* test.

Data were analysed as means and standard deviations (SD) and reported in millimetres. Differences were reported as absolute values. To assess the reproducibility of the thermoforming process (P vs. P2 and A vs. A2) the differences in thicknesses between the thermoformed as-received aligners were assessed by means of Student’s *t* test for paired data (significance level after Bonferroni correction for multiple comparison was set at *p* < 0.0014) and Dahlberg’s error. The changes after intraoral use for both P and A (T1 vs. T0) were assessed by means of Student’s *t* test for paired data (significance level after Bonferroni correction for multiple comparison was set at *p* < 0.0014). Standard Statistical Software Package (SPSS version 22.0, SPSS IBM, Armonk, NY, USA) and Microsoft Excel 365 (Microsoft Office 365, Microsoft Corporation, Redmond, WA, USA) were used for the statistical analysis.

## Results

All the patients showed satisfactory adherence to the treatment, and all the retrieved aligners were used for the study.

The method error ranged between 0.02 and 0.07 mm for all thickness measurements, and there was no systematic error for any of the measurements analysed (Student’s *t* test, *p* > 0.05).

Before intraoral use, the average thickness of P ranged from 0.38 ± 0.08 (U6DPR) to 0.69 ± 0.04 mm (U6DPL and U6MPL), while the average thickness of A ranged from 0.42 ± 0.09 (U6DPR) to 0.68 ± 0.04 mm (U6DPL) (Table 1).

**Table 1:** Mean (± SD) aligner thickness in millimetres of the as-received clear aligners after duplication of the thermoforming process

	P		P2		P-P2				A		A2		A-A2			
Point	Mean (mm)	SD	Mean (mm)	SD	Mean (mm)	SD	*p* value	DAHL (mm)	Mean (mm)	SD	Mean (mm)	SD	Mean (mm)	SD	*p* value	DAHL (mm)
U3R	0.51	0.10	0.50	0.10	0.06	0.06	0.684	0.06	0.55	0.10	0.56	0.07	0.07	0.06	0.508	0.06
U4BR	0.45	0.09	0.47	0.09	0.05	0.05	0.316	0.04	0.50	0.10	0.53	0.08	0.07	0.06	0.239	0.07
U4PR	0.47	0.10	0.46	0.10	0.04	0.04	0.820	0.04	0.51	0.09	0.50	0.08	0.06	0.03	0.466	0.05
U5BR	0.43	0.09	0.44	0.10	0.05	0.05	0.527	0.05	0.48	0.10	0.50	0.08	0.06	0.05	0.301	0.06
U5PR	0.42	0.11	0.42	0.11	0.06	0.05	0.842	0.06	0.47	0.11	0.47	0.09	0.06	0.03	0.973	0.05
U6MBR	0.43	0.09	0.44	0.10	0.05	0.04	0.779	0.05	0.48	0.10	0.49	0.09	0.06	0.04	0.517	0.05
U6MPR	0.40	0.09	0.41	0.10	0.05	0.04	0.685	0.05	0.46	0.09	0.46	0.11	0.06	0.04	0.929	0.05
U6DBR	0.40	0.10	0.40	0.09	0.05	0.04	0.972	0.13	0.46	0.10	0.46	0.08	0.07	0.04	0.745	0.13
U6DPR	0.38	0.08	0.39	0.10	0.05	0.04	0.600	0.05	0.42	0.09	0.43	0.13	0.07	0.06	0.640	0.07
U3L	0.68	0.06	0.63	0.07	0.06	0.06	0.011	0.06	0.66	0.05	0.65	0.04	0.06	0.03	0.349	0.04
U4BL	0.65	0.06	0.62	0.05	0.05	0.03	0.010	0.04	0.64	0.05	0.64	0.06	0.05	0.04	0.971	0.05
U4PL	0.65	0.06	0.64	0.04	0.04	0.03	0.253	0.03	0.64	0.04	0.65	0.04	0.04	0.03	0.675	0.03
U5BL	0.68	0.04	0.64	0.05	0.05	0.03	0.003	0.04	0.66	0.03	0.65	0.04	0.03	0.03	0.379	0.03
U5PL	0.66	0.04	0.64	0.03	0.04	0.02	0.161	0.03	0.67	0.05	0.67	0.03	0.04	0.03	0.717	0.03
U6MBL	0.67	0.04	0.66	0.04	0.03	0.02	0.190	0.02	0.67	0.03	0.66	0.04	0.03	0.03	0.175	0.03
U6MPL	0.69	0.04	0.66	0.05	0.03	0.02	*0.001*	0.02	0.66	0.05	0.67	0.03	0.03	0.03	0.412	0.03
U6DBL	0.65	0.06	0.64	0.05	0.04	0.04	0.492	0.04	0.67	0.04	0.66	0.05	0.03	0.02	0.059	0.02
U6DPL	0.69	0.04	0.67	0.03	0.04	0.03	0.116	0.03	0.68	0.04	0.67	0.04	0.03	0.03	0.768	0.03

*P* passive aligner, *A* active aligner, *DAHL* Dahlberg’s error. Absolute values are reported in P-P2 and A-A2. Statistically significant results (P vs. P2 and A vs. A2) are reported in italics

Concerning the dimensional reproducibility of the thermoforming process (Table 1), overall good reproducibility was observed for both P and A aligners. With regard to P, statistically significant differences between P and P2 were only found at the point U6MPL (*p* = 0.001). Furthermore, the highest error was found at U6DBR (Dahl = 0.13 mm). Regarding A, no statistically significant difference between A and A2 was detected in any of the measured points. The highest error was found at U6DBR (Dahl = 0.13 mm).

Concerning the thickness changes of the aligners after 10 days of full-time intraoral use, at T1, the thickness of P ranged from 0.44 ± 0.11 (U6DBR) to 0.65 ± 0.09 mm (U6MPL), while the thickness of A ranged from 0.43 ± 0.12 (U6DPR) to 0.67 ± 0.04 mm (U6DPL) (Table 2). The intra-group comparison (T1 vs. T0) showed statistically significant difference only at U3L (*p* = 0.001) for P, while no statistically significant differences were observed in the intra-group comparison for the eighteen measured points of the A aligner (Table 2).

**Table 2:** Mean (± SD) aligner thickness in millimetres of the as-received clear aligners (T0) and retrieved clear aligners after 10 days of intraoral use (T1)

	P							A						
	T0		T1		T1-T0			T0		T1		T1-T0		
Point	Mean (mm)	SD	Mean (mm)	SD	Mean (mm)	SD	*p* value	Mean (mm)	SD	Mean (mm)	SD	Mean (mm)	SD	*p* value
U3R	0.51	0.10	0.49	0.09	0.07	0.03	0.165	0.55	0.10	0.56	0.07	0.09	0.06	0.206
U4BR	0.45	0.09	0.47	0.09	0.07	0.06	0.537	0.50	0.10	0.53	0.08	0.08	0.08	0.690
U4PR	0.47	0.10	0.47	0.10	0.10	0.07	0.985	0.51	0.09	0.50	0.08	0.08	0.06	0.388
U5BR	0.43	0.09	0.46	0.10	0.07	0.07	0.310	0.48	0.10	0.50	0.08	0.09	0.07	0.182
U5PR	0.42	0.11	0.44	0.09	0.09	0.09	0.666	0.47	0.11	0.47	0.09	0.10	0.09	0.784
U6MBR	0.43	0.09	0.46	0.13	0.09	0.10	0.206	0.48	0.10	0.49	0.09	0.10	0.08	0.553
U6MPR	0.40	0.09	0.45	0.14	0.09	0.09	0.127	0.46	0.09	0.46	0.11	0.10	0.09	0.850
U6DBR	0.40	0.10	0.44	0.11	0.07	0.07	0.049	0.46	0.10	0.46	0.08	0.11	0.08	0.583
U6DPR	0.38	0.08	0.45	0.12	0.09	0.11	0.043	0.42	0.09	0.43	0.12	0.09	0.09	0.766
U3L	0.68	0.06	0.61	0.11	0.08	0.06	*0.001*	0.66	0.05	0.65	0.04	0.04	0.04	0.106
U4BL	0.65	0.06	0.62	0.09	0.05	0.04	0.023	0.64	0.05	0.64	0.06	0.04	0.02	1.000
U4PL	0.65	0.06	0.61	0.08	0.07	0.05	0.071	0.64	0.04	0.65	0.04	0.04	0.02	0.504
U5BL	0.68	0.04	0.62	0.10	0.08	0.07	0.009	0.66	0.03	0.65	0.04	0.03	0.02	0.598
U5PL	0.66	0.04	0.63	0.08	0.05	0.06	0.091	0.67	0.05	0.67	0.03	0.04	0.02	0.638
U6MBL	0.67	0.04	0.64	0.09	0.07	0.07	0.140	0.67	0.03	0.66	0.04	0.04	0.03	0.446
U6MPL	0.69	0.04	0.65	0.09	0.05	0.07	0.196	0.66	0.05	0.67	0.03	0.04	0.03	0.199
U6DBL	0.65	0.06	0.62	0.09	0.08	0.06	0.192	0.67	0.04	0.66	0.05	0.04	0.03	0.746
U6DPL	0.69	0.04	0.63	0.13	0.07	0.11	0.158	0.68	0.04	0.67	0.04	0.01	0.01	1.000

*P* passive aligner, *A* active aligner. Absolute values are reported in T1-T0. Statistically significant results (T1 vs. T0) are reported in italics

## Discussion

The present clinical prospective study aimed to assess the surface thickness changes of orthodontic clear aligner (CA) in two different settings: due to the thermoforming process and after the physiological intraoral exposure. The results suggest that, in terms of thickness variation, the thermoforming process adopted for this study showed good reproducibility; also, the intraoral ageing did not determine clinically relevant changes that can affect the efficacy of the orthodontic aligners.

In the current study, all the objectives were studied analysing two aligners configurations: passive (no movement, no shape for attachment) and active (tooth movement, shapes for attachments and divot). This approach aimed to evaluate whether the presence of modification shapes of the aligners for attachments and divots interfered with the accuracy of the thermoforming process. Interestingly, slightly greater inaccuracies were found in the thermoforming of the passive aligners, as compared with that of the active one, thus suggesting that the presence of aligner auxiliaries did not influence the reproducibility of the thermoforming process in terms of dimensional changes. In addition, the number of thickness changes reported with a statistically significant difference did not correspond to a clinically relevant thickness difference. Interestingly, the thickness of both P and A resulted in inhomogeneity throughout the aligner occlusal surface, ranging from a minimum value of 0.38 mm to a maximum value of 0.69 mm. Also, compared to the original thickness of the thermoplastic foil used for the aligner manufacturing (0.75 mm), on average, the occlusal surface resulted in reduced thickness at all the reference points adopted in the current study. However, this dimensional variation seems to be constant whenever the same aligner is thermoformed twice, supporting a good reproducibility of the thermoforming process.

This study was the first to assess the dimensional stability of orthodontic CA in terms of thickness changes of the entire occlusal surface of the device after in vivo intraoral exposure. It is crucially important to measure thickness changes of the occlusal surface as they might be influenced not only by factors that can be easily studied in in vitro experiments, such as temperature and humidity, but also by different oral functional and parafunctional activities (chewing, talking, drinking, swallowing, clenching or grinding). Due to the difficulties in monitoring the intraoral environment, the previous researches assessing the mechanical and dimensional proprieties of orthodontic thermoplastic materials were performed in a simulated intraoral environment with artificial saliva and/or on fragments or specimens of retrieved aligners [13, 18, 20–22]. The methodology of the current study allowed to analyse the surface dimension of the entire aligner at different reference points, overtaking the limitations related to the not uniform structure of the aligner.

With regard to the effects of intraoral ageing, the results of the present studies suggested that the degree of deformation of the CA surface was slightly greater in the passive aligner, as compared with the active aligner. Since there were no differences in the materials, thickness, and thermoforming process of the active and the passive aligners, this result might be explained by other external factors related to the environment where the aligners were used. One possible explanation is that, since in the current study the passive aligner was always the first aligner wore by the patients, a more intense masticatory muscle activity might be present during a first adaptation phase, thus influencing the wear of the aligner [34, 35].

The results of the current study should be interpreted with caution as 10 days of intraoral use have been studied. With regard to the active orthodontic treatment with CA, change of the orthodontic aligners varies from 7 to 14 days according to the clinicians’ indication; therefore, differences in the number of days wearing the appliance might influence the number of thickness changes. Also, due to the limited time in the intraoral environment, the results of the current study cannot be extended to the same thermoplastic materials used for retention devices, which are permanently used in the intraoral environment after the end of the active orthodontic treatment [36].

One previous study by Ryokawa and colleagues examined the mechanical properties of flat thermoplastic material specimens in a simulated intraoral condition, as well as the thickness changes after thermoforming and water absorption [20]. The authors observed a thickness increase due to water absorption ranging from 100.3 to 119.9%, supporting the hypothesis that the mechanical properties of the thermoplastic materials vary due to external factors, such as intraoral temperature changes and the presence of saliva. However, the study of the intraoral ageing of orthodontic materials plays a crucial role in the current orthodontic literature since a great variety of potential ageing factors can alter the morphologic and structural characteristics of polymeric and metallic materials [23]. Therefore, the complexity of the intraoral environment cannot be easily reproduced in in vitro experimental settings. Furthermore, when studying the intraoral ageing of orthodontic materials, other individual factors related to the mastication activity should be taken into account, such as the variability of the masticatory muscle activity and the differences in the degree of oral parafunctional behaviours [34, 37]. Therefore, retrieval analysis is considered a first step in approaching the complex intraoral interaction pattern.

Previous studies assessed the structure of aligners after intraoral exposure, pointing out substantial morphological variations relative to the as-received specimens involving microcracks, abrasion at the cusp tips, adsorption of integuments and localised calcification of the precipitated biofilm at stagnation sites [22, 27]. Differently from the current study, the authors adopted scanning electron microscopy and energy dispersive X-ray microanalysis on aligner fragments. Also, in the abovementioned studies, the aligners were worn for 14 days, instead of 10, and different thermoplastic materials were used.

The choice of the appropriate aligner thickness is a crucial issue in the manufacturing process of orthodontic aligners [25]. The importance of the thickness of the aligners is related to the amount of load exerted on the periodontal ligament and to the orthodontic force delivered to each tooth in order to obtain the planned sequential dental movements. As a matter of fact, studies on one of thinnest commercially available aligners (0.5-mm thick) pointed out relevant overloading of teeth which might impact periodontal structures [29]. Similarly, one research assessing the influence of the aligner material type and thickness on the force delivery proprieties pointed out that thin materials exerted higher energy than thicker materials of the same brand [24]. In particular, to investigate the effects of thickness changes, Kwon and co-authors adopted differences in thickness of about 0.2 mm. The results of the current study showed thickness changes around 0.05 mm, both after thermoforming and after intraoral ageing, which although statistically significant are not likely to determine clinically relevant changes in the force delivery system. These results allow to speculate that the aligners adopted in the current study present a stable geometrical structure in the in vivo environment. However, these results must be ascribed to the specific materials (PET-G), thickness (0.75 mm) and production procedures adopted in the current study for the aligner manufacturing, as different materials might respond differently to both thermoforming and intraoral ageing.

Since several factors contribute to the behaviour of the force system during intraoral use, further studies are needed to determine the actual impact of these very small thickness changes on the mechanical behaviour, the treatment efficacy and the clinical performance of the CA studied.

## Conclusion

The thermoforming process reduces the thickness of the aligners, compared to the original dimension of the thermoplastic foil. However, in terms of occlusal surface thickness, it is a reproducible process both for passive and active aligner configurations. Therefore, the thermoforming process is not influenced by modification shape of the aligners (attachments or divot).

Also, within the limits of the current study, after 10 days of full-time intraoral use, non-relevant thickness alterations of the aligner occlusal surface were observed, thus suggesting a good dimensional stability of the analysed orthodontic aligners during treatment.

## Data Availability

The datasets used and/or analysed during the current study are available from the corresponding author on reasonable request.

## Abbreviations

A: Active aligner
CA: Clear aligners
P: Passive aligner
PET-G: Polyethylene terephthalate glycol copolyester
